# Downregulation of HLA Class I molecules in the tumour is associated with a poor prognosis in patients with oesophageal squamous cell carcinoma

**DOI:** 10.1038/sj.bjc.6604715

**Published:** 2008-10-07

**Authors:** Y Mizukami, K Kono, T Maruyama, M Watanabe, Y Kawaguchi, K Kamimura, H Fujii

**Affiliations:** 1 First Department of Surgery, University of Yamanashi, 1110 Shimokato, Chuo-city, Yamanashi 409-3898, Japan

**Keywords:** HLA class I antigen, oesophageal squamous cell carcinoma, prognosis

## Abstract

As antigenic peptides in the context of human leukocyte antigen (HLA) class I molecules are recognised by cytotoxic T lymphocytes (CTL), the downregulation of HLA class I molecules is one of the reasons why tumour cells can evade CTL-mediated anti-tumour immunity. In this study, we investigated HLA class I expression in oesophageal squamous cell carcinoma (ESCC) (*n*=70) and in their metastatic lesions (lymph nodes (*n*=40) and liver (*n*=3)), by immunohistochemistry with anti-HLA class I monoclonal antibody (EMR8-5). As a result, the downregulation of HLA class I expression in primary lesions of ESCC was observed in 43%, and that in metastatic lymph nodes was noted in 90%. Furthermore, patients with preserved HLA class I expression in primary tumours showed a better survival in comparison to those with downregulated HLA class I molecules (*P*<0.01). Furthermore, multivariate analysis using Cox's proportional hazards model revealed that the downregulated expression of HLA class I in primary lesions was an independent, unfavourable prognostic factor (*P*<0.01). In conclusion, the downregulation of HLA class I expression frequently occurred in primary tumour and, to a greater extent, in metastatic lesions of patients with ESCC and was associated with patient survival.

Oesophageal squamous cell carcinoma (ESCC) is one of the most lethal malignancies (Shimada *et al*, 2003). Despite aggressive treatment modalities such as surgical tumour resection with extensive lymphadenectomy and chemo-radiation therapy, the long-term disease control of advanced-stage ESCC remains difficult (Akiyama *et al*, 1994; Ando *et al*, 2003; Kleinberg and Forastiere, 2007). Therefore, immunotherapy such as the utilisation of anti-tumour T cells or antibodies induced by cancer vaccination is extremely appealing. As the anti-tumour cytotoxic T lymphocyte (CTL) response is induced by the recognition of immunogenic epitopes that are presented on various types of HLA class I molecules on the tumour (Marincola *et al*, 2000; Tangri *et al*, 2001; Cabrera *et al*, 2007; Nagorsen and Thiel, 2008), it is important to evaluate the status of HLA class I molecules on tumour cells. It has been reported that the downregulation of HLA class I molecules in the tumour commonly occurred, and the defect of HLA class I molecules is significantly related to patient survival in several malignancies including ESCC (Hosch *et al*, 1997a; Kageshita *et al*, 1999; Ryschich *et al*, 2005; Vitale *et al*, 2005; Ogino *et al*, 2006; Ramnath *et al*, 2006; Speetjens *et al*, 2008; Ueda *et al*, 2008). Moreover, it is well known that the downregulation of HLA class I on the tumour allows it to evade CTL-mediated anti-tumour immunity, leading to variant cancer cells that arise from the parent tumour during tumour progression at both primary and metastatic sites.

Although immunohistochemical analyses of HLA class I expression in several malignancies have previously been performed, there was a discrepancy in the frequencies of downregulated HLA class I molecules (Mattijssen *et al*, 1991; Möller *et al*, 1991; Passlick *et al*, 1996; Vora *et al*, 1997; Ramnath *et al*, 2006; Kikuchi *et al*, 2007; Mehta *et al*, 2008; Speetjens *et al*, 2008). This discrepancy might be due to a difficulty in evaluating immunostaining by anti-MHC class I monoclonal antibodies (mAbs), such as W6/32, HC-10, or HC-A2, as these anti-MHC class I mAbs were not appropriate for the immunostaining of formalin-fixed, paraffin-embedded tissue. Recently, EMR8-5, which is an mAb against HLA class I heavy chains (HLA-A,B,C), has been confirmed to be valid in HLA class I immunohistochemistry (Tsukahara *et al*, 2006; Kikuchi *et al*, 2007; Kitamura *et al*, 2007).

To our knowledge, there have been few reports describing HLA class I expression in ESCC analysed by previously available mAbs (Hosch *et al*, 1997a; Nie *et al*, 2001). Thus, in this study, we investigated HLA class I expression in primary and metastatic lesions (lymph node and liver) in ESCC patients by immunohistochemistry using EMR8-5 mAbs. Furthermore, we evaluated the correlation between HLA class I expression and the clinicopathological status or clinical outcome in patients with ESCC.

## Materials and methods

### Patients and samples

Seventy patients with oesophageal squamous cell carcinoma, who were operated on in the University of Yamanashi Hospital between April 5, 1994, and July 27, 2004, were enrolled in this study. Follow-up duration of the patients was from 49 to 168 months. The characteristics of the study subjects are summarised in Table 1. None of the patients received radiotherapy, chemotherapy, or other medical interventions before the study. This study was approved by the Ethical Committee of the University of Yamanashi, and written informed consent was obtained from all individuals.

### Immunohistochemical analysis

Four-micrometre-thick sections of archival, formalin-fixed, paraffin-embedded tissue blocks (ESCC and adjacent normal oesophagus) were used for immunohistochemical analysis. For HLA class I staining, the sections were deparaffinized, followed by antigen retrieval with epitope retrieval solution (10 mmol citrate buffer (pH 6.0), Dakocytomation) in an autoclave (121°C, 20 min). Endogenous peroxidase was blocked by chemmate peroxidase blocking solution (Dakocytomation). The primary antibody EMR8-5 (anti-HLA class I heavy chain, diluted by PBS, 1 : 100, Cosmo Bio Co., Tokyo, Japan) was applied to the sections at 4°C overnight. Thereafter, the sections were incubated with the streptavidin–biotin complex (Simple Stain MAX-PO kit, Nichirei, Tokyo, Japan) for 30 min. The sections were then treated with 3, 3′-diaminobenzidine (Dakocytomation) for 5 min, and counterstained with haematoxylin. Normal epithelium, stromal cells, or lymphocytes served as a positive control. Negative control staining was performed with isotype control mAbs (Dakocytomation).

For the evaluation of HLA class I expression, two independent observers (YM and KK) assessed HLA class I positivity semi-quantitatively, without previous knowledge of clinicopathological data. The intensity of HLA class I staining was evaluated using the following criteria: strong positive (strong), dark brown staining in more than 50% of tumour cells completely obscuring cytoplasm; weak positive (weak), any lesser degree of brown staining appreciable in tumour cells; absent, no appreciable staining in tumour cells.

### Statistical analysis

Actuarial overall survival rates were analysed by the Kaplan–Meier method, and survival was measured in days from the operation to death or the last review. Differences between survival curves were analysed by the log-rank test. Deviation between the population classified by the intensity of HLA class I expression and clinicopathological factors was evaluated by the *χ*^2^ test.

To assess the correlation between survival time and multiple clinicopathological variables, univariate and multivariate analyses were conducted using Cox's proportional hazards model. Differences were considered significant at *P*<0.05. All statistical analyses were performed with StatView-J 5.0 software (Abacus Concepts, Berkeley, CA, USA).

## Results

### Immunohistochemical analysis of HLA class I expression in ESCC

**Figure 1:**
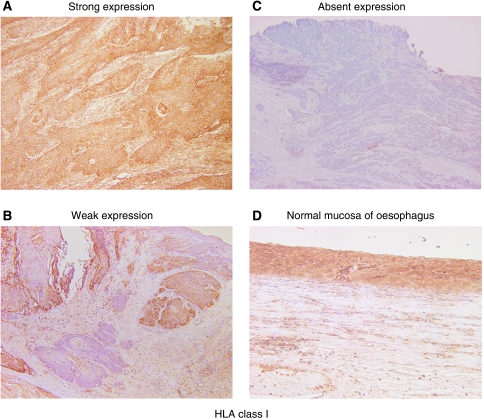
Representative immunohistochemical staining of MHC class I molecules in primary lesions of ESCC patients. The intensity of HLA class I staining was evaluated using the following criteria: strong expression, dark brown staining in more than 50% of tumour cells completely obscuring the cytoplasm (**A**); weak expression, any lesser degree of brown staining appreciable in tumour cells (**B**); absent expression, no appreciable staining in tumour cells (**C**). Positive control for HLA class I expression involving normal oesophageal mucosa (**D**).

The intensity of HLA class I staining was semi-quantified by immunohistochemical staining into strong, weak, and absent expressions, evaluated by the criteria described in Materials and Methods, and representative immunostainings are shown in Figure 1. Summarised data from ESCC (*n*=70) indicated that preserved HLA class I expression (strong) in the primary tumour was seen in 40 out of 70 patients, weak expression in 24 out of 70 patients, and absent expression in 6 out of 70 patients (Table 2).

The frequencies of the downregulation of HLA class I expression in the primary tumour were not distributed equally in relation to the disease progression, such as stage and tumour factors (Table 2).

### HLA class I expression between primary tumour and metastatic site

**Figure 2:**
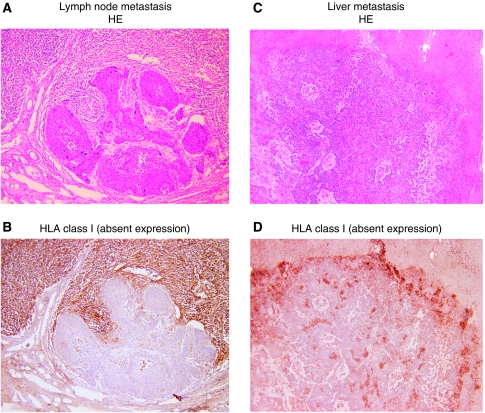
Representative immunohistochemical staining of MHC class I molecules in metastatic lymph nodes (**B**) and the liver (**D**) of ESCC patients. Serial sections for haematoxylin–eosin (HE) staining are shown in (**A**) and (**C**).

Out of the total 70 patients, there were 40 with lymph node metastasis and 3 with hepatic metastasis (1 synchronous and 2 metachronous metastases). Representative immunostainings for HLA class I molecules in lymph node and hepatic metastases are shown in Figure 2. As presented in Table 3, 36 (90%) out of 40 patients with lymph node metastasis revealed a downregulation of HLA class I expression. In particular, even if the primary tumour exhibited strong HLA class I expression (*n*=21), most of the lymph node metastases (*n*=17, 81%) showed the downregulation of HLA class I expression, indicating that the downregulation of HLA class I expression frequently occurred according to lymph node metastasis.

Similarly, all hepatic metastases showed the downregulation of HLA class I expression, although the number of cases was limited (*n*=3).

Of note, a total loss (absent) of HLA class I expression was noted in 28% of lymph node and 67% of hepatic metastases.

### HLA class I expression relating to survival in ESCC patients

**Figure 3:**
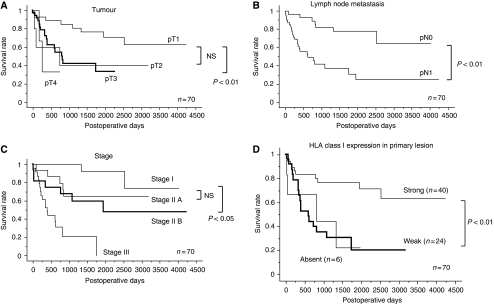
Survival curves in ESCC patients. Kaplan–Meier analyses of the survival of patients with ESCC according to variables are demonstrated as T factors (**A**), lymph node metastasis (**B**), stage (**C**), and HLA class I expression in primary lesions (**D**), respectively (*n*=70, *P*-values by the log-rank test). (**D**) HLA class I expression in primary lesions was classified into strong (*n*=40), weak (*n*=24), or absent (*n*=6). (**A**) The median survival time in pT1 was significantly longer than that in pT3, but there were not significant differences in any other combination. (**B**) The median survival time in pN0 was significantly longer than that in pN1. (**C**) The median survival time in Stage I was significantly longer than that in Stage II B, but there were not significant differences in any other combination. (**D**) The median survival time in strong expression was significantly longer than that in weak expression in terms of HLA class I expression.

To investigate the relationship between HLA class I expression and the clinical outcome, we analysed patient survival relating to HLA class I expression in the primary tumour. As shown in Figure 3D, the patients showing the downregulation of HLA class I (weak or absent) in the primary tumour showed a significantly poorer prognosis than patients with preserved HLA class I expression (strong), as well as several other factors, such as T factor (Figure 3A), *N* factor (Figure 3B), and stage classification (Figure 3C). Moreover, among the metastatic lymph node cases (*n*=40), there was not significant difference in the overall survival between the cases with further downregulation of HLA class I status in lymph node metastasis (*n*=22) and the remaining 18 cases with consistent class I status between primary and metastatic lesions (data not shown).

To further assess whether HLA class I expression in the primary tumour represented a prognostic parameter in patients with ESCC, we used Cox's proportional hazards model. The covariate parameters included several clinicopathological factors in addition to HLA class I, as shown in Table 4. On univariate analysis, several factors including HLA class I showed a significantly higher hazard ratio for a poor prognosis. Moreover, when multivariate analysis was performed using significant factors in univariate analysis, multivariate analysis revealed that only HLA class I expression was an independent prognostic factor (*P*<0.01, Table 4). These results clearly indicated that the downregulation of HLA class I expression in the primary tumour was closely related to a poor prognosis.

## Discussion

In this study, the downregulation of HLA class I molecules in the primary tumour was seen in 43% of ESCC (30 out of 70 cases), and the frequency of downregulated HLA class I in the metastatic lymph nodes (90%) was more greatly increased than that in the primary tumour. In addition, the downregulation of HLA class I expression in the primary tumours was an independent prognostic factor showing a higher hazard ratio for a poor prognosis.

In this study, the downregulation of HLA class I was seen in 43% of ESCCs, in line with previous reports (41–45%) involving analysis using different mAbs for oesophageal cancer (Rockett *et al*, 1995; Hosch *et al*, 1997b). It is most likely that the downregulation of HLA class I frequently occurred in ESCC, as the downregulation of HLA class I has been reported at a rate of 10–50% among various types of cancer (Garrido *et al*, 1997).

This is the first study, to our knowledge, reporting that the frequency of downregulated HLA class I in the metastatic lymph nodes was much higher (90% of the case) than that in primary lesions. Comparative analysis between the primary tumour and metastasis in the same patient indicated that even if the primary tumour showed a strong expression of HLA class I molecules, tumour cells in the lymph node metastasis revealed the downregulation of HLA class I. Thus, a loss or downregulation of HLA class I within the primary tumour might be one of the mechanisms whereby tumour cells spread from primary lesions, resulting in the establishment of lymph node metastasis.

It has been demonstrated that human tumours with various histologies have low or downregulated HLA class I molecules due to the modulation or inhibition of the expression of various HLA class I antigen-processing machinery (APM) components (Seliger, 2008). Four different phenotypes of altered HLA class I molecules are known, which are as follows: (i) total HLA loss; (ii) HLA haplotype loss; (iii) HLA locus loss; and (iv) HLA allelic loss. The mechanisms involved in the downregulation of HLA class I have been reported to be genetic mutation or the suppressed transcriptional activity of the *HLA class I heavy chain or β-2 microglobulin*, mutation of the transporter associated with antigen-processing (*TAP*) gene, or inhibition of the transport of HLA class I molecules (Seliger, 2008).

It is well known that abnormality of HLA class I molecules and APM in tumour cells is one of the major reasons for escape from CD8(+) cytotoxic T cells, resulting in disease progression (Seliger, 2008). However, it has also been shown that tumour cells without HLA class I molecules appear susceptible to NK cell-mediated killing due to the involvement of killer-cell inhibitory receptors on the surface of NK cells, indicating that tumour cells with a total loss of HLA class I might be killed by NK cell-mediated immunity (Seliger, 2008). Moreover, tumour cells showing the downregulation of specific HLA class I alleles could escape from T-cell-mediated immunity and also avoid NK cell-mediated killing due to sufficient HLA class I expression (Garrido *et al*, 1997). These observations suggest that there is a complex association between the altered HLA class I expression in the tumour and T-cell or NK cell-mediated immunity. Further detailed studies are required to evaluate the phenotype of altered HLA class I molecules in ESCC.

In this study, the downregulation of HLA class I expression indicated an independent prognostic factor associated with a poor prognosis in patients with ESCC, in line with a previous report (Hosch *et al*, 1997b). Furthermore, we recently reported a significant correlation between the positivity of peptide-specific T-cell responses to cancer-testis antigens and preserved HLA class I expression in primary lesions of ESCC (Mizukami *et al*, 2008). These observations suggested that T-cell-mediated immunity related to HLA class I expression may affect the prognosis of patients with ESCC. As the downregulation of HLA class I expression frequently occurred in ESCC, the preservation of HLA class I expression on tumours might be one of the inclusion criteria for cancer vaccination therapy for ESCC. Furthermore, the treatment strategy aiming at restoring HLA class I expression might be able to improve survival among patients with ESCC or might lead to successful immunotherapy.

In conclusion, we showed that the downregulation of HLA class I expression frequently occurred in primary tumours and, to a greater extent, in metastatic lesions of patients with ESCC and was associated with patient survival.

## Figures and Tables

**Table 1:** Patient and tumour characteristics (*n*=70)

Age (years, mean±s.d.)	64.9±8.9
	
*Gender*	
Male : female	66 : 4
*Tumour size (mm)*	
Mean (±s.d.)	48.2±21.6
	
*Tumour* a	
T1	28
T2	5
T3	34
T4	3
	
*Lymph node metastasis* a	
N0	30
N1	40
	
*Histological classification*	
Well	15
Moderately	38
Poorly	17
	
*Stage* a	
I	14
IIA	16
IIB	16
III	24
IV	0

Abbreviations: Moderately=moderately differentiated squamous cell carcinoma; poorly=poorly differentiated squamous cell carcinoma; well=well-differentiated squamous cell carcinoma.

a Tumour, lymph node metastasis, and stage according to the TNM classification for oesophageal cancer (UICC).

**Table 2:** HLA class I expression of tumours in relation to clinicopathologic status

	**HLA class I expression in primary lesion**			
	**Strong (*n*=40)**	**Weak (*n*=24)**	**Absent (*n*=6)**	
*Stage* a				
I (*n*=14)	12	1	1	
IIA (*n*=16)	7	8	1	^**^
IIB (*n*=16)	11	2	3	
III (*n*=24)	10	13	1	
				
*Tumour* a				
T1 (*n*=28)	22	3	3	
T2 (*n*=5)	1	3	1	^**^
T3 (*n*=34)	15	17	2	
T4 (*n*=3)	2	1	0	
				
*Lymph node metastasis* a				
N0 (*n*=30)	19	9	2	
N1 (*n*=40)	21	15	4	NS
				
*Histological classification*				
Well (*n*=15)	7	8	0	
Moderately (*n*=38)	27	10	1	^**^
Poorly (*n*=17)	6	6	5	

Abbreviations: Moderately=moderately differentiated squamous cell carcinoma; NS=not significant; poorly=poorly differentiated squamous cell carcinoma; Well=well-differentiated squamous cell carcinoma.

^**^P<0.05 by the *χ*^2^ test.

a Tumour, lymph node metastasis, and stage according to the TNM classification for esophageal cancer (UICC).

**Table 3:** (A) Correlation of HLA class I expression between primary lesion and lymph node metastasis (*n*=40); (B) Correlation of HLA class I expression between primary lesion and liver metastasis (*n*=3)

	**Lymph node metastasis**		
**Primary lesion**	**Strong (*n*=4)**	**Weak (*n*=25)**	**Absent (*n*=11)**
Strong (*n*=21)	4	15	2
Weak (*n*=15)	0	10	5
Absent (*n*=4)	0	0	4
		**Liver metastasis**	
		**Weak (*n*=25)**	**Absent (*n*=11)**
Strong (*n*=2)		1	1
Weak (*n*=1)		0	1
Absent (*n*=0)		0	0

Abbreviations: Absent=absent expression; strong=strong expression; weak=weak expression.

**Table 4:** Univariate and multivariate analyses of patients

		**Univariate analysis**		**Multivariate analysis**	
		**Overall survival**		**Overall survival**	
**Variables**	**Categories**	**HR (95% CI)**	***P*-value**	**HR (95% CI)**	***P*-value**
Age (years)	<65 (*vs* ⩾65)	1.76 (0.85–3.62)	0.13		
Gender	Male (*vs* female)	1.80 (0.25–13.21)	0.56		
Tumour size (mm)	⩾45 (*vs* <45)	2.51 (1.20–5.26)	0.014^**^	1.81 (0.72–4.54)	0.21
Tumour^a^	T3+T4 (*vs* T1+T2)	2.73 (1.25–5.95)	0.012^**^	1.18 (0.44–3.14)	0.74
Lymph node metastasis^a^	N1 (*vs* N0)	4.48 (1.91–10.55)	<0.01^**^	2.86 (0.82–10.03)	0.10
Stage^a^	Stage II+III (*vs* stage I)	6.55 (1.55–27.73)	0.011^**^	1.31 (0.22–7.83)	0.77
HLA class I expression	Weak+absent (*vs* strong)	4.03 (1.87–8.70)	<0.01^**^	3.42 (1.40–8.33)	<0.01^**^
Lymphatic invasion	Positive (*vs* negative)	3.18 (1.34–7.52)	<0.01^**^	1.03 (0.32–3.34)	0.96
Vascular invasion	Positive (*vs* negative)	2.74 (1.31–5.75)	<0.01^**^	1.48 (0.58–3.80)	0.42
Histological classification	Poorly (*vs* well+moderately)	1.67 (0.78–3.57)	0.18		

Abbreviations: HR=hazard ratio; 95% CI=95% confidence interval; moderately=moderately differentiated squamous cell carcinoma; poorly=poorly differentiated squamous cell carcinoma; well=well-differentiated squamous cell carcinoma.

^**^P<0.05.

a Tumour, lymph node metastasis, and stage according to the TNM classification for oesophageal cancer (UICC).
